# Italian Adults’ Likelihood of Getting COVID-19 Vaccine: A Second Online Survey

**DOI:** 10.3390/vaccines9030268

**Published:** 2021-03-17

**Authors:** Luigi Roberto Biasio, Guglielmo Bonaccorsi, Chiara Lorini, Daniela Mazzini, Sergio Pecorelli

**Affiliations:** 1 Giovanni Lorenzini Foundation, Viale Piave 35, 20129 Milan, Italy; sergio.pecorelli@lorenzinifoundation.org; 2 Department of Health Sciences, University of Florence, 50134 Florence, Italy; guglielmo.bonaccorsi@unifi.it (G.B.); chiara.lorini@unifi.it (C.L.); 3 Central Tuscany Local Health Unit, 50134 Florence, Italy; daniela.mazzini@uslcentro.toscana.it

**Keywords:** COVID-19, online surveys, vaccines, vaccine literacy

## Abstract

Rapid online surveys are an important tool for tracking the public’s knowledge and perceptions during infectious disease outbreaks. In June 2020, during the early phases of COVID-19 vaccines development, we conducted a survey in 885 Italian adults that aimed at assessing their attitudes and opinions about vaccination, in addition to their vaccine literacy levels (i.e., skills in finding, understanding, and using information about vaccines). In January 2021, the same questionnaire was administered to a similar population (*n* = 160). Interactive vaccine literacy was significantly higher in January 2021 than in June 2020 (mean score 3.38 vs. 3.27 respectively, *p* = 0.0021). The percentage of participants willing to be vaccinated against COVID-19 assessed by either-or questions, was equally high in both surveys (>90%), which is quite reassuring, although metrics based on categorical scales cannot identify hesitant subjects.

## 1. Introduction

Since the onset of the COVID-19 pandemic, various surveys have been conducted aimed at assessing people’s vaccine acceptance, showing significant differences across countries, ranging from about 40 to >90% [1]. These discrepancies may be due to different causes, including methodologies used to collect data: most of the investigations were self-reported, conducted via the web, some were longitudinal, and others were cross-sectional. Moreover, in some studies, answers were gathered, forcing the respondents to express an either-or opinion (nominal scales), while others used odd-numbered ordinal scales, such as Likert’s. The latter allows for a more sensitive evaluation of attitudes, also identifying hesitant individuals, and produce a better distribution of data, but may be less objective, leaving the possibility of intermediate or indecisive answers. Findings from these kind of tools are useful to guide communication strategies and information campaigns by public health authorities to counter vaccine hesitancy, but may provide little help when specific individuals’ opinions are key to comprehending their instant behavior.

In June 2020, we conducted a rapid online cross-sectional survey [2] to evaluate the feasibility of assessing the levels of health literacy skills about vaccination (vaccine literacy) in 885 Italian adults. Other objectives were to collect opinions and attitudes of the interviewees about candidate COVID-19 vaccines, including the willingness to get vaccinated, using nominal scales (“yes” or “no” questions). Results showed that the intention to receive a COVID-19 vaccine was very high (92%) and significantly greater than receiving seasonal influenza vaccine (66%). This investigation also showed quite high levels of functional and interactive-critical vaccine literacy, i.e., individuals’ abilities to collect, understand, comprehend and use information about vaccines.

Following conditional marketing authorization for some COVID-19 vaccines granted by the European Medicines Agency (EMA) in December 2020, the survey was repeated targeting a similar population, at a time when vaccination campaigns started in Italy. The study aimed to verify whether opinions and attitudes had changed, in particular about the likelihood of vaccine acceptance. A secondary objective was to assess vaccine literacy skills using a validated scale.

## 2. Materials and Methods

In January 2021, we conducted a cross-sectional survey using the same methodology as for a previous one (June 2020) [2]. An anonymous online questionnaire, to which the respondents (>18 years of age) could choose to complete or not, was prepared, distributed, and collected by SurveyMonkey. The same questions used in the first investigation were administered, except for the items regarding COVID-19 and flu vaccines, due to situational changes. All variables of the questionnaire administered in June 2020 are listed in Table 1, while the updated items are reported in Table 2. Assessing the participants’ willingness to get a COVID-19 vaccine (measured by a nominal scale) was the primary objective of this new survey. To address the secondary objective (i.e., to assess vaccine literacy skills), we used an already validated psychometric scale [3], including four functional and eight interactive-critical items. Answers were rated on a forced 4 point Likert scale for frequency. As in previous studies using a similar scale [2,4,5], the score was obtained from the mean value of the answers to each item (range 1 to 4), a higher value corresponding to a higher vaccine literacy level.

We distributed the questionnaire using the same methods as the previous survey, adopting a convenience sample. An URL, linking to the questionnaire, was posted via Facebook or sent via e-mail to the same addressees of the previous survey, selected from the mailing list of Giovanni Lorenzini Foundation (Milan, Italy), asking them to forward the link to others without communicating back their list of the addressees. A reminder was sent one week later. The addressees were balanced according to three geographical areas, northern, central, and southern Italy & the largest islands (Sardinia and Sicily).

The questionnaire was composed of two pages: on the first page, participants were provided with information about the rationale and scope of the survey, i.e., to gather opinions and attitudes as well as modalities and abilities to collect, understand, and use information about vaccination, including COVID-19 vaccines. Respondents were asked to provide honest answers, were not given any incentives for participation, and could reply only once to the survey. They were informed that proceeding to the second page of the survey and completing the questionnaire constituted consent. No targeted replies were purchased. Participants could send answers via computers, tablet, or smartphone.

Statistical analysis was carried out using MedCalc Statistical Software (Ostend Belgium) version 18.2.1 [6] by means of descriptive tables including percentages, means, standard deviations (SD), confidence intervals (CI), medians, and non-parametric tests, as the data did not follow a normal distribution. In particular, Mann–Whitney test for independent variables was used to compare the results of the new survey vs. the previous one. Kruskal–Wallis test was applied to assess the association between variables. Cronbach’s alfa coefficient was calculated to confirm the internal consistency of the collected data of vaccine literacy skills. For each analysis, an alpha level = 0.05 was considered as significant.

Considering the results of the previous survey, assuming a prevalence of 8% refusal, at 95% confidence level and 5% confidence interval, 114 responses were considered to be the minimum acceptable number.

## 3. Results

Answers were collected instantly, right at the time when COVID-19 vaccination campaign started in Italy, from 12 January to 30 January 2021; 160 respondents completed and submitted the questionnaire.

The main findings are reported in Table 3. Cronbach’s coefficient values, calculated from the replies to both the functional and interactive-critical scales, were acceptable (=0.8030 and =0.7029, respectively) and comparable to those of the previous survey.

### 3.1. Demographics

The demographic composition of the participants in the second survey were not completely representative of the first one. Fifty-six percent of participants were in the 18–30 age class (in the preceding survey they were 23%), the remaining were evenly distributed between 31 and 65 years of age; only two persons were over 65. The difference among age groups was highly significant (Mann–Whitney *p* < 0.001). Females accounted for 62%, while in June 2020 they were 50% (Mann–Whitney *p* = 0.006)

Ninety-eight percent of participants were native Italian speakers.

Differences between areas of residence and educational degree were not statistically significant, while the occupational status showed marked differences, with a higher percentage of students (43%), with respect to the first survey (14%) (Mann–Whitney *p* < 0.001) and much less employed and retired persons. Information sources were very similar in both investigations, with the highest preference for internet and streaming (81%), followed by TV (47%) and social media (40%).

### 3.2. Vaccine Literacy, Attitudes, and Opinions

Regarding vaccine literacy skills, scores were comparable between the two surveys for both the functional and the interactive-critical scales, although the latter was significantly higher in January 2021 (score 3.38 ± 0.46 vs. 3.27 ± 0.54, Mann–Whitney *p* < 0.05).

Results were also similar when comparing all participants aged less than 65 years, including the higher interactive-critical score from the second survey, even though not significant. Moreover, a significant association was observed in both surveys between a lower interactive-critical score and younger participants (18–30 yrs) with respect to older ages (Kruskal–Wallis *p* < 0.05), while there were no significant differences among age groups for the functional score and the willingness to be vaccinated.

In January 2021, 91% of respondents intended to receive one of the COVID-19 vaccines, the majority (89%) trusting their safety and efficacy. However, 50% of interviewees believed that the characteristics of various vaccines do not overlap with each other, and 61% would prefer to choose which one to receive. Fifty-nine percent of respondents were in favor of a mandatory COVID-19 vaccination, and 69% considered the Government capable of offering the vaccine to everyone for free, whereas many (79%) were willing to pay for the shot, and 69% percent believed that children should be immunized too. Differently than in the previous survey, in January 2021 female participants showed a significant higher willingness to be vaccinated with respect to males (Kruskal–Wallis *p* < 0.05).

Regarding seasonal flu immunization, 38% of respondents had been vaccinated against flu during the last seasonal campaign. In addition, 21% didn’t succeed in receiving the shot due to vaccine shortage. Sixty-five percent stated that they intended to be immunized against other infectious diseases, in addition to COVID-19 and influenza.

The majority of respondents disagreed completely with both statements: *‘I am not favorable to vaccines because they are unsafe’* (77%) and *‘There is no need to vaccinate because natural immunity exists’* (82%). Few respondents were partially in disagreement (20% and 14%, respectively), and much fewer were partially in agreement (3% and 4%, respectively). Answers in total agreement with both statements were very rare (<1%). These proportions were not significantly different from the precedent survey. Noteworthy, positive opinions about vaccines for both statements were significantly associated with higher interactive-critical vaccine literacy levels (Kruskal–Wallis *p* < 0.05), likelihood to accept COVID-19 (*p* < 0.001) and flu vaccination (*p* < 0.05), but not with functional vaccine literacy, any of the age classes, gender, education or occupational status.

## 4. Discussion

### 4.1. Findings and Comparison to Previous Survey

From the results of the survey conducted in January 2021, using the same methodology, the percentage of respondents willing to be vaccinated (91%) was very similar to that observed in June 2020, when vaccines were still in Phase 1 and 2 of the clinical development. Yet, in the second survey there were much fewer respondents (*n* = 160) and significant disproportions between age groups and gender, although we targeted the same population. However, this has not influenced substantially the interpretation of the main results (willingness to be vaccinated and vaccine literacy scores), as the associations of these variables with the different age groups remained similar in both studies.

Individuals may also have improved their ability to understand and use information as a result of the infodemic: this appears to be confirmed by the higher interactive-critical vaccine literacy levels observed with respect to the previous investigation, while the functional skills (i.e., simply gathering information) were similar.

Noteworthy, this recent survey confirms that in Italy the vast majority of respondents trust the safety and efficacy of the COVID-19 vaccines that have recently been authorized. Also relevant is the proportion of individuals available to pay a fee to get immunized. These are reassuring findings for the future vaccine uptake, although about a half of participants believe that the characteristics of vaccines do not overlap with each other and would prefer to choose which one to receive. These observations also confirm that, among those who stated a willingness to be vaccinated, there are hesitations and doubts, which are likely to increase because of the quantity and complexity of information available about the efficacy and safety of vaccines coming into use, which is often very technical and contradictory.

Regarding flu immunization, only 38% were vaccinated, albeit this might be linked to the mean young age of the respondents and to the fact that some of the participants were not able to get vaccinated because of the vaccine shortage during the recent flu immunization campaign.

Also relevant is the observation that most of the opinions about vaccination were positive, although some respondents were just partially in disagreement with the statements considering vaccines unsafe and useless. The significant association of positive opinions with interactive-critical vaccine literacy levels confirms the relevance of people’s abilities in understanding and comprehending information about vaccination.

### 4.2. Comparison to Other Research

An investigation carried out via the web in Italy in September 2020 has shown that only 54% would have accepted receiving a COVID-19 vaccine [7]. On the contrary, we observed a high proportion (>90%) who were willing to get vaccinated in both surveys.

Large variability in COVID-19 vaccine acceptance rates has been reported worldwide, varying from 40% up to >90% [1,8,9]. Moreover, recent surveys have shown, over time, a reduction of the acceptance, probably linked to the decreasing trust in information from the media—often contradictory—and in governmental communication. In a US longitudinal panel survey, self-reported likelihood of getting a COVID-19 vaccine declined from 74% in April to 56% in December 2020, despite the press releases of high vaccine efficacy for two mRNA vaccines, prior to emergency use authorization granted from the FDA [10].

Discrepancies between results may be linked to population diversities, geographical situations, time of execution of the studies, in addition to the different methodologies adopted for data collection. Interestingly, the proportion of individuals unwilling to be vaccinated (13%) during the first week of our survey in June 2020, was similar to that shown in another inquiry of 1004 adults conducted in Italy a few days before, in May 2020, using a 5 point Likert scale. In this study, 41% of the participants declared to be unwilling (15%) or hesitant (26%) towards COVID-19 vaccines [11,12]. This investigation has been repeated in December 2020, showing a similar proportion (16%) of individuals refusing to get vaccinated [13]. From our survey in June, the intention to be vaccinated improved in the second week of data collection, from 88% to 96%, along with a significant increase in positive perceptions about candidate (at that time) vaccines. This corresponded, time-wise, to the announcement (13 June 2020), largely reported by the media, of the agreement between the Europe’s Inclusive Vaccines Alliance (IVA) and a vaccine manufacturer to supply massive doses of vaccine, starting by the end of 2020.

### 4.3. Limitations

The main limitations of the study were the small sample of the population and the disproportion between age groups of respondents from 2020 and 2021.

Regarding the low number of participants, repeated cross-sectional studies have a greater possibility of losing respondents with respect to longitudinal panel surveys. In addition, the number of surveys currently proposed on the web, as well as the saturation of the public with the huge amount of contradictory news about the pandemic may have a negative impact on the number of respondents, in particular when using a convenience sampling, instead of recruiting participants by professional panel providers.

Other limits of the study were common to most of the online surveys and related to low participation of people with lower educational levels [14] and the elderly: in Italy, only 42% of individuals between 65 and 74 y of age surf the web, compared to almost 90% of the 18–50 yrs classes [15]. Moreover, self-reported metrics may not correlate with future behavior, in particular for small samples of the population [10].

### 4.4. Future Research

Achieving acceptable vaccination coverage against SARS-CoV-2 and herd immunity is still long and difficult, and will be characterized by an increasing amount of information that may enhance cognitive and emotional overload in the population. Online surveys will continue to have an important role in better addressing communication and counter vaccine hesitancy. All methodologies used to collect and analyze data may be useful in future research, depending on the different objectives of studies.

## 5. Conclusions

Rapid online surveys are an important tool in tracking the public’s knowledge and perceptions during infectious disease outbreaks, especially when face-to-face research is restricted due to control measures. Opinions and attitudes of the respondents to a survey conducted in January 2021, using either-or questions, were positive despite the small sample size, and similar to those shown during the early phases of the clinical development of COVID-19 vaccines, with >90% willing to get vaccinated. Despite the low number of participants these findings are reassuring. However, clear communication strategies and educational campaigns remain necessary to counter hesitancy and maintain the public’s confidence.

## Figures and Tables

**Table 1:** June 2020 survey—questions used to assess skills, perceptions, attitudes, and opinions.

Variable	Measure and Items	Assessment/Score
Vaccine Literacy functional skills	When reading or listening to information about future COVID-19 vaccines or current vaccines: Did you find words you didn’t know? Did you find that the texts were difficult to understand? Did you need much time to understand them? Did you or would you need someone to help you understand them?	Ordinal, 4 points Likert scale for frequency: Often (1), Sometimes (2), Rarely (3), Never (4)
Vaccine Literacy interactive/ critical skills	When looking for information about future COVID-19 vaccines or current vaccines: Have you consulted more than one source of information? Did you find the information you were looking for? Have you had the opportunity to use the information? Did you discuss what you understood about vaccinations with your doctor or other people? Did you consider whether the information collected was about your condition? Have you considered the credibility of the sources? Did you check whether the information was correct? Did you find any useful information to make a decision on whether or not to get vaccinated?	Ordinal, 4 points Likert scale for frequency: Often (4), Sometimes (3), Rarely (2), Never (1)
Opinions about vaccination	How much do you agree with the following statements: ‘I am not favorable to vaccines because they are unsafe’ ‘There is no need to vaccinate because natural immunity exists’	Ordinal, 4 points Likert scale for agreement: Totally (1), A little (2), Partially(3), Not at all (4)
COVID-19 vaccines perceptions and attitudes	About future COVID-19 vaccines: Will be possible to produce safe and efficacious vaccines? Will you get vaccinated, if possible? Will Authorities succeed in vaccinating the entire population? Would you pay a fee to be vaccinated? Should children be vaccinated too?	Nominal YES/NO
Current vaccines attitudes	About current vaccines: Were you vaccinated against flu last season? Will you get vaccinated against flu this year? Do you plan to be vaccinated against other infectious diseases?	Nominal YES/NO

**Table 2:** January 2021 survey—questions used to assess attitudes about COVID-19 and flu vaccines.

**Variable**	**Measure and Items**	**Assessment**
COVID-19 vaccines attitudes	About COVID-19 vaccines: Do you think the vaccines developed so far are safe? Do you think they are efficacious? Do you think they overlap, regardless of the production technique used? Do you intend to get vaccinated against COVID-19? If you could, would you choose which vaccine to take? Will the Government be able to offer the vaccine against COVID-19 for everyone for free? Would you pay a fee to be vaccinated? Should vaccination against COVID-19 be made mandatory for everyone? Should vaccination against COVID-19 be made compulsory for the most at-risk groups? Do you think children should be vaccinated too?	Nominal YES/NO
Current vaccines attitudes	About other vaccines: Have you been vaccinated against seasonal flu? Did you want to be vaccinated against the flu, but you couldn’t? Have you been recently vaccinated and/or do you intend to be vaccinated soon against other infectious diseases, in addition to seasonal influenza and COVID-19?	Nominal YES/NO

**Table 3:** Main demographics, vaccine literacy (VL) scores and attitudes toward vaccinations in the total populations and in participants aged < 65 years. Findings from the June 2020 and January 2021 surveys and level of significance.

Variable		June 2020 *n* = 885 *(<65 yrs n = 803)*	January 2021 *n* = 160 *(<65 yrs n = 158)*	*p* (*)
**Sex (F %)**		50%	62%	=0.006
**Age classes**	18–30 yrs	206 (23%)	89 (56%)	<0.001
	31–50 yrs	327 (37%)	36 (23%)	
	51–65 yrs	270 (31%)	33 (20%)	
	>65 yrs	82 (9%)	2 (1%)	
**Educational degree (§)**	Secondary	356 (40%)	51 (32%)	n.s.
	Tertiary	478 (54%)	102 (64%)	
	Others	21 (6%)	7 (4%)	
**Residence area**	Northern	260 (30%)	42 (26%)	n.s.
	Central	455 (53%)	103 (65%)	
	Southern	140 (17%)	14 (9%)	
**Functional VL mean score** **(SD) [95% CI]**		2.92 (0.70) [2.87–2.97]	2.99 (0.63) [2.89–3.08]	n.s.
***Functional VL mean score*** ***Participants < 65 yrs of age (SD) [95% CI]***		2.92 (0.70) [2.87–2.97]	2.99 (0.63) [2.89–3.08]	n.s.
**Interactive-critical VL mean score** **(SD) [95% CI]**		3.27 (0.54) [3.23–3.30]	3.38 (0.46) [3.23–3.30]	=0.021
***Interactive-critical VL mean score*** ***Participants < 65 yrs (SD) [95% CI]***		3.28 (0.53) [3.25–3.32]	3.38 (0.46) [3.30–3.45]	*n.s.*
**Willing receiving COVID-19 vaccine**		816 (92%)	145 (91%)	n.s.
***Willing receiving COVID-19 vaccine*** ***Participants < 65 yrs***		743 (93%)	143 (91%)	*n.s.*
**Planning/receiving seasonal flu vaccine**		588 (66%)	95 (59%)	n.s.
***Planning/receiving seasonal flu vaccine*** ***Participants < 65 yrs***		516 (64%)	93 (59%)	*n.s.*
**Planning receiving other vaccines**		649 (73%)	104 (65%)	n.s.
***Planning receiving other vaccines*** ***Participants < 65 yrs***		590 (73%)	102 (65%)	*n.s.*

(*) = Mann–Whitney for independent samples, margin of error 5%; CI = confidence interval; n.s. = not statistically significant). (§) = Tertiary education = college, university, master; secondary education = high schools, professional schools; others = primary, lower secondary schools.

## Data Availability

Data supporting reported results are available upon request to the corresponding author.
